# Spiritual needs among hospitalized patients at a public hospital in Brazil: a cross-sectional study

**DOI:** 10.1590/1516-3180.2021.0985.R2.19052022

**Published:** 2022-08-29

**Authors:** Cassio Murilo Trovo Hidalgo, Ana Julia Aguiar de Freitas, Lucas Salviano de Abreu, Hendrio Reginaldo Santiago, Alessandro Gonçalves Campolina

**Affiliations:** I MD. Resident, Internal Medicine Department, Hospital do Servidor Público Municipal (HSPM), São Paulo (SP), Brazil.; II BSc. Doctoral Student, Molecular Oncology Research Center, Barretos Cancer Hospital, Teaching and Research Institute, Barretos (SP), Brazil.; III MD. Resident, Internal Medicine Department, Hospital do Servidor Público Municipal (HSPM), São Paulo (SP), Brazil.; IV MD. Resident, Internal Medicine Department, Hospital do Servidor Público Municipal (HSPM), São Paulo (SP), Brazil.; V MD, MSc, PhD. Scientific Researcher, Centro de Investigação Translacional em Oncologia, Instituto do Câncer do Estado de São Paulo (ICESP), Faculdade de Medicina da Universidade de São Paulo (FMUSP), São Paulo (SP), Brasil.

**Keywords:** Palliative care, Spirituality, Religion, Brazil, SNAP, Sao Paulo, Spiritual distress, Observational study, Spiritual needs

## Abstract

**BACKGROUND:**

The relationship between spirituality and health has been the object of growing discussion. There is a lack of data on spiritual needs assessments in Brazil.

**OBJECTIVE:**

This study aimed to assess the spiritual needs of patients admitted to a public tertiary hospital and perform a comparative analysis between patients with and without indications for palliative care.

**DESIGN AND SETTING:**

A cross-sectional observational study included patients hospitalized between August and December 2020 in Hospital do Servidor Publico Municipal, Sao Paulo, Brazil.

**METHODS:**

The included patients answered a questionnaire consisting of sociodemographic data, the Duke religiosity scale, and the Spiritual Needs Assessment for Patients (SNAP) tool for a spiritual needs assessment. The World Health Organization Palliative Needs tool (NECPAL) was used to evaluate the indications for palliative care. The level of significance adopted was 5%.

**RESULTS:**

A total of 66 patients were included in this study. Most participants (97%) declared themselves as belonging to a religion. The group without indication for palliative care by the NECPAL showed greater spiritual (P = 0.043) and psychosocial needs (P = 0.004). No statistically significant difference was observed in the religious needs domain (P = 0.176). There were no statistically significant differences in the Duke scale scores between the two groups.

**CONCLUSION:**

Spiritual, psychosocial, and religious needs are prevalent among hospitalized patients, and multidisciplinary teams must consider these needs in their management approach. In addition, this study suggests that psychosocial and spiritual needs can be even higher in patients who do not receive palliative care.

## INTRODUCTION

The relationship between spirituality and health has been the subject of growing discussion and study. Spirituality has long been related only to religion; however, its definition has expanded to include what is sacred and gives the final purpose to life.^1,2^

Spirituality can also be understood as a human propensity to seek meaning in life through concepts that transcend the tangible. Its association with health has become a paradigm to be established in daily medical practice since disease remains an entity with a broad impact on clinical approaches.^3^

Religious and spiritual beliefs have proven to aid in coping with the most diverse situations of imbalance and the health of individuals as a preparation for death.^4^

Diagnosis of life-threatening conditions can lead to spiritual suffering. Most patients with life-threatening health conditions have reported the importance of spirituality.^5^ This demonstrates that patients' beliefs are increasingly related as a protective factor against the development of high emotional stress throughout the diagnosis.^6^ The benefits of spirituality, including treatment adherence and resilience of patients living with HIV,^7^ chronic kidney disease,^8^ heart failure,^9^ and cancer, have been studied in several populations.^10^

Conversely, patients with unmet spiritual demands may experience compromised care.^11^ This condition is also a predictive factor for worse quality of life in patients with advanced chronic diseases.^12^ Despite the evidence regarding the influence of spiritual well-being in the disease process, published data on this subject remain scarce.

Several tools have been developed to identify patients experiencing spiritual distress. A review showed at least eight validated questionnaires assessing spiritual needs.^13^ Cultural diversity may influence the results obtained from different populations. Therefore, the Spiritual Needs Assessment for Patients (SNAP) scale, culturally adjusted and translated into Portuguese, is the primary tool validated for assessing spiritual needs in Brazil.^14,15^

Interest in spiritual care is growing worldwide, including in Brazil. However, there is a lack of data on spiritual needs in hospitalized patients.^16^ In this context, this study proposes identifying spiritual needs in different domains using the adapted SNAP scale.

## OBJECTIVE

This study aimed to assess the spiritual needs of patients admitted to a public tertiary hospital and to conduct a comparative analysis between patients with and without indications for palliative care.

## METHODS

### Study design

This observational cross-sectional study aimed to assess the spiritual needs of hospitalized patients at the Hospital do Servidor Publico Municipal (HSPM) in Sao Paulo, Brazil.

The patients hospitalized between August and December 2020 were included. The inclusion criteria consisted of patients who were at least 18 years old, voluntarily provided written informed consent, and were capable of understanding, interpreting, and answering the questionnaires. Patients who could not complete the questionnaires or had impaired consciousness were excluded.

### Data collection

The data were obtained through questionnaires administered during face-to-face interviews conducted by the author, coauthors, and the research volunteers. Patients were randomly selected through a draw, and questionnaires were administered from August to December 2020. The questionnaire consisted of three main parts. The first part included clinical and sociodemo-graphic data evaluating variables such as age, sex, marital status, and aspects of the patient's primary diagnosis. Next, patients were assessed for religiosity using a version of the Duke religiosity scale (DUREL) validated in Brazil, consisting of organizational, non-organizational, and intrinsic religiosity domains.^17^ The third part was the assessment of spiritual needs using the SNAP scale, using a version adapted for a Brazilian population.^15^ This questionnaire evaluates the patient through three subscales: psychosocial (5 items), spiritual (13 items), and religious (5 items), with objective questions to quantify the patient's needs in each respective domain. Patients were divided into two groups according to whether palliative care was indicated by the World Health Organization Palliative Needs tools (NECPAL) in its adapted form for Brazilian culture.^18,19^ The palliative performance score (PPS) was evaluated for every patient in this study.

### Statistical analysis

For sample size calculation, an effect size of 0.66 was considered. In addition, the probabilities of type I and type II errors were set to 0.05 and 0.20, respectively. Thus, a sample of 31 cases per group was calculated, with a total of 62 cases. The Wilcoxon-Mann-Whitney test was used to compare means.

Descriptive analyses of the data were carried out using tables containing absolute values and proportions (in the case of qualitative variables), mean, median, standard deviation (SD), and quartiles (in the case of quantitative variables). The Chi-square and Fisher's exact tests were used for categorical variables comparisons. For comparisons between categorical and numerical variables, the T-test and analysis of variance (ANOVA) (in the case of data showing normality) or Mann–Whitney and Kruskal–Wallis tests (in the non-parametric context) were used. The level of significance adopted was 5%. Statistical analyses were performed using the IBM SPSS Inc. (version 18.0) package for Windows (IBM, Chicago, United States, 2009).

### Ethical considerations

This study was approved by the Research Ethics Committee of HSPM (protocol number 29728920.0.0000.5442) on August 13, 2020. This project complies with the Declaration of Helsinki (1964 and later versions of 1975, 1983, 1989, 1996, 2000, and 2008) and Resolution No. 466 of 2012 of the National Health Council. The study details were adequately explained, and informed consent was obtained from each participant.

## RESULTS

A total of 66 patients were included in the study, with a mean hospital stay of 11.3 days (standard deviation [SD] = 15.5). Among the participants, 65.2% were female, and 34.8% were male. The mean age was 60.1 years, ranging from 21 to 84 years (SD = 15.3). The predominant marital status in the study population was married (39.4%), while 28.8% of the participants were widowed, 16.7% single, and 10.6% divorced. The majority (97%) declared themselves as belonging to one or more religions, the most prevalent being Catholic and Evangelical (33.3%), and spiritism was also prevalent (13.6%). Most patients were hospitalized for clinical conditions (81.8%), surgical causes accounted for 18.2% of the sample, and the mean length of stay was 11.3 days (SD = 15.1). The most frequent diagnoses of the studied population were neoplasms (37.9%), cardiovascular and cerebrovascular diseases (25.8%), and gastrointestinal and liver disorders (13.7%). Participants were classified as with or without an indication for palliative care according to the NECPAL tool. There were 33 (50%) participants in each group. The mean PPS was 69.8 (SD = 25). The demographic data are detailed in Table 1.

**Table 1:** Clinical and demographic data of patients included in the study's analysis. Sao Paulo, Brazil, 2020

		n	%
**Sex**	Female	43	65.2%
	Male	23	34.8%
**Marital status**	Married	26	39.4%
	Widowed	19	28.8%
	Single	11	16.7%
	Divorced	7	10.6%
	Stable union	3	4.5%
**Work**	Yes	27	40.9%
	No	39	59.1%
**Retired**	Yes	39	59.1%
	No	27	40.9%
**Public Server**	Yes	48	72.7%
	No	18	27.3%
**Type of hospitalization**	Clinical	54	81.8%
	Surgical	12	18.2%
**Main diagnosis**	Neoplasm	25	37.9%
	Cardiovascular and cerebrovascular diseases	17	25.8%
	Gastrointestinal tract diseases	9	13.7%
	Pulmonary diseases	4	6.0%
	Infectious conditions	4	6.0%
	Other	4	6.0%
	Kidney diseases	3	4.6%
**PC indication**	Yes	33	50.0%
	No	33	50.0%
**Religion**	Yes	64	97.0%
	No	2	3.0%
**Religious practice**	Catholic	22	33.3%
	Evangelical	22	33.3%
	Spiritist	9	13.6%
	Believes in God without a religion	6	9.2%
	Buddhist	3	4.6%
	Umbanda	2	3.0%
	Catholic and Spiritist	2	3.0%

PC = palliative care.

**Figure 1:**
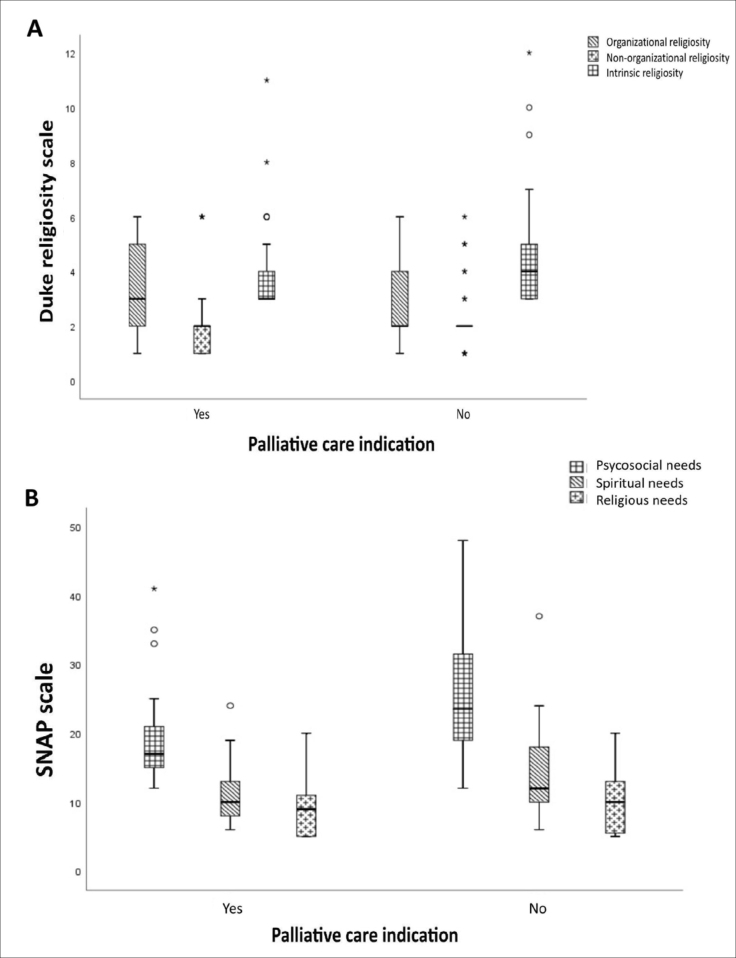
Domain scores from the Duke scale (**A**) and Spiritual Needs Assessment for Patients (SNAP) questionnaire (**B**), according to the indication of palliative care follow-up. Sao Paulo, Brazil, 2020.

The Duke and SNAP scores for each group are represented graphically in Figures 1A and 1B, respectively. When comparing patients between groups with and without indications for palliative care, a statistically significant difference was observed, indicating greater spiritual (P = 0.043) and psychosocial (P = 0.004) needs in the groups that had no indication for follow-up palliative care. The religious need variable was not statistically significant between the groups (P = 0.176). We did not observe any statistical differences in the Duke scale scores between the two groups (Table 2).

**Table 2:** Comparative analysis of religiosity and spiritual needs between groups with and without indications for palliative care follow-up. Sao Paulo, Brazil, 2020

		PC indication		P value
		Yes	No	
**Organizational religiosity (Duke)**	Mean	3.25	2.82	0.427
	SD	1.83	1.42	
	Median	3.00	2.00	
	Minimum	1.00	1.00	
	Maximum	6.00	6.00	
**Non-organizational religiosity (Duke)**	Mean	2.25	2.45	0.169
	SD	1.55	1.25	
	Median	2.00	2.00	
	Minimum	1.00	1.00	
	Maximum	6.00	6.00	
**Intrinsic religiosity (Duke)**	Mean	4.03	4.52	0.242
	SD	1.77	2.17	
	Median	3.00	4.00	
	Minimum	3.00	3.00	
	Maximum	11.00	12.00	
**Psychosocial needs (SNAP)**	Mean	19.00	25.00	0.004
	SD	6.74	9.15	
	Median	17.00	23.50	
	Minimum	12.00	12.00	
	Maximum	41.00	48.00	
**Spiritual needs (SNAP)**	Mean	11.12	14.28	0.043
	SD	4.27	6.59	
	Median	10.00	12.00	
	Minimum	6.00	6.00	
	Maximum	24.00	37.00	
**Religious needs (SNAP)**	Mean	9.03	10.59	0.176
	SD	4.29	4.87	
	Median	9.00	10.00	
	Minimum	5.00	5.00	
	Maximum	20.00	20.00	

PC = palliative care; SD = standard deviation; SNAP = Spiritual Needs Assessment for Patients.

## DISCUSSION

This study used the SNAP tool to assess spiritual needs in a sample of hospitalized patients in the public health system in Brazil. The results showed a greater need for psychosocial and spiritual domains in patients who did not meet the NECPAL criteria for palliative care follow-up.

To fulfill the spiritual needs using a medical approach, it is necessary to recognize the importance of this topic. People experience deep and wide-ranging demands in life according to different personal contexts. In particular, psychosocial and spiritual needs become more evident in the illness process. Data suggest that patients in Brazil are often not assessed for religiosity and spirituality. In most cases, physicians are unaware of the patient's religion and spiritual values, which could lead to spiritual distress and inadequate assistance.^20^

Studies have shown that spiritual needs can be related to variables such as the time of diagnosis and stage of the disease, showing the need for an individualized approach to spiritual care.^21^ This care is often directed at patients with chronic illnesses in end-of-life care. However, patients with acute conditions that are potentially life-threatening bring spiritual needs that require validation and a professional approach.^22^

In this context, palliative care professionals should be able to approach the spiritual suffering of hospitalized patients. A model of spiritual care by trained professionals that seeks to obtain a focused spiritual history and screen for possible unmet demands, involving cultural limits and individual values of family members and patients is suggested.^23^ Training healthcare professionals in spiritual care is a promising strategy for a holistic approach, as suggested by a Brazilian study.^24^ The inclusion of spiritual care in the continuing education of these professionals can benefit a greater number of patients who do not have access to specialized palliative care staff to meet their spiritual needs.

Vilalta et al. suggested that awareness of the reality of the disease may be a beneficial factor for spiritual needs to be met.^25^ A reason for the findings in our study is that patients who have an indication for palliative care are approached more frequently about their health condition and, therefore, have greater knowledge about the disease, thus finding the resilience to face the illness process.

Finally, it is important to note that this study has some limitations. As an observational analytical study, it was impossible to evaluate the impact of the differences in the spiritual needs of the patients analyzed. Regarding the small sample with a heterogeneous population, factors such as the time of diagnosis and severity of the current disease may be confounding factors in the data analysis.

As most published studies on spiritual care focus only on patients with advanced chronic illnesses and considering the lack of studies addressing this topic in Brazil, further research is suggested. This should involve larger samples and focus on the hospitalized population, using spiritual assessment tools to evaluate the need for spiritual intervention regardless of health conditions.

## CONCLUSION

Spiritual, psychosocial, and religious needs are prevalent among hospitalized patients, and a multidisciplinary team must consider these needs in their management approaches. This study suggests that according to the SNAP scale, psychosocial and spiritual needs can be even higher in patients who are not under palliative care.
